# The Role of BiP Retrieval by the KDEL Receptor in the Early Secretory Pathway and its Effect on Protein Quality Control and Neurodegeneration

**DOI:** 10.3389/fnmol.2017.00222

**Published:** 2017-07-17

**Authors:** Hisayo Jin, Mari Komita, Tomohiko Aoe

**Affiliations:** ^1^Department of Anesthesiology, Graduate School of Medicine, Chiba University Chiba, Japan; ^2^Department of Anesthesiology, Chiba Rosai Hospital Ichihara, Japan; ^3^Pain Center, Chiba Medical Center, Teikyo University Ichihara, Japan

**Keywords:** ER (endoplasmic reticulum) stress, KDEL receptor, BiP, chaperones, neurodegeneration

## Abstract

Protein quality control in the early secretory pathway is a ubiquitous eukaryotic mechanism for adaptation to endoplasmic reticulum (ER) stress. An ER molecular chaperone, immunoglobulin heavy chain-binding protein (BiP), is one of the essential components in this process. BiP interacts with nascent proteins to facilitate their folding. BiP also plays an important role in preventing aggregation of misfolded proteins and regulating the ER stress response when cells suffer various injuries. BiP is a member of the 70-kDa heat shock protein (HSP70) family of molecular chaperones that resides in the ER. Interaction between BiP and unfolded proteins is mediated by a substrate-binding domain and a nucleotide-binding domain for ATPase activity, leading to protein folding and maturation. BiP also possesses a retrieval motif in its carboxyl terminal. When BiP is secreted from the ER, the Lys-Asp-Glu-Leu (KDEL) receptor in the post-ER compartments binds with the carboxyl terminal KDEL sequence of BiP and returns BiP to the ER via coat protein complex I (COPI) vesicular transport. Although yeast studies showed that BiP retrieval by the KDEL receptor is not essential in single cells, it is crucial for multicellular organisms, where some essential proteins require retrieval to facilitate folding and maturation. Experiments in knock-in mice expressing mutant BiP with the retrieval motif deleted revealed a unique role of BiP retrieval by the KDEL receptor in neuronal development and age-related neurodegeneration.

## Introduction

The endoplasmic reticulum (ER) is the first organelle of the secretory pathway and plays an important role in synthesizing proteins and the lipid membrane. Newly synthesized polypeptides are inserted into the ER via translocon (Alder et al., 2005). Nascent peptides bind to ER molecular chaperones such as immunoglobulin heavy chain-binding protein (BiP/GRP78), calnexin, calreticulin and protein disulfide isomerase present in the ER lumen, thereby escaping aggregation and degradation. These proteins are modified with sugar chains and disulfide bonds to form a three-dimensional structure and complexes with other protein subunits (Zhang et al., 1997). Proteins with mature tertiary structure are separated from molecular chaperones, transported from the ER to the Golgi, and further secreted into other cellular compartments such as the endosome and plasma membrane or secreted extracellularly (Ellgaard and Helenius, 2003).

Immature (unfolded or misfolded) proteins are formed due to extrinsic insults (e.g., ischemia, malnutrition, hypoxia and toxic substances) or intrinsic insults (e.g., mutated sequence, missing subunits). They accumulate in the ER, inducing the ER stress response or unfolded protein response (UPR). The UPR comprises three major events: (1) increased molecular chaperone production (Yoshida et al., 2001); (2) general translational repression of proteins (Harding et al., 1999); and (3) decomposition of unfolded proteins by the ubiquitin-proteasome system (ER-associated protein degradation, ERAD; Plemper et al., 1997).

**Figure 1:**
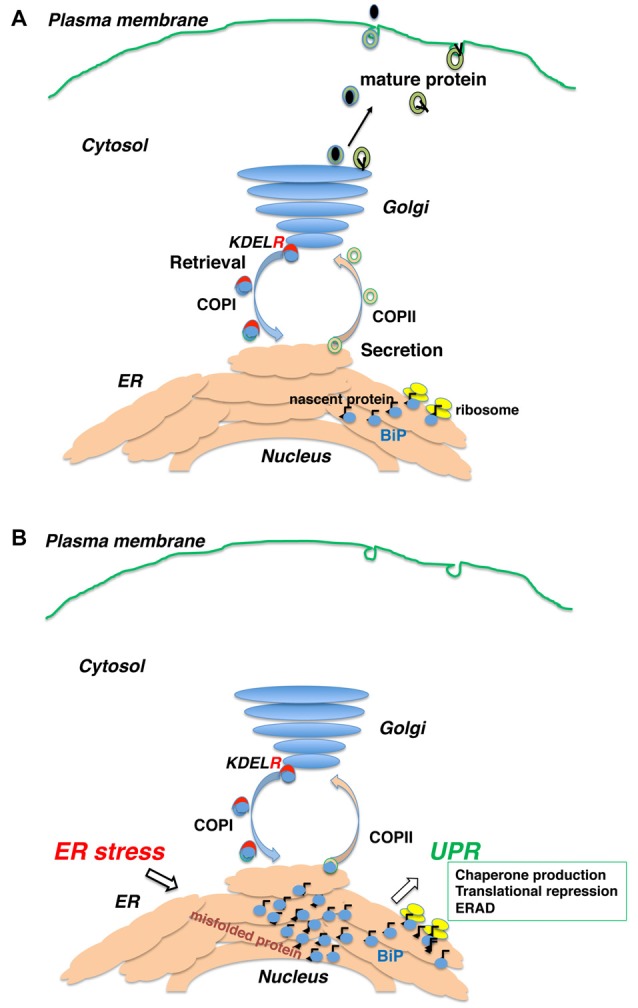
Newly synthesized polypeptides are inserted into the endoplasmic reticulum (ER) and transported to the secretory pathway. **(A)** Secretory proteins and membrane proteins are inserted into the ER via the translocon, where they associate with ER molecular chaperones. Proteins with mature structures are transported from the ER to the Golgi and further secreted into the plasma membrane or extracellularly. **(B)** Immature (unfolded or misfolded) proteins due to intrinsic defects (e.g., mutated sequence, missing subunits) or extrinsic insults (e.g., ischemia, malnutrition, hypoxia and toxic substances) accumulate in the ER, inducing the ER stress response or unfolded protein response (UPR).

The UPR expands the capacity for quality control to compensate for accumulation of unfolded proteins in the ER (Feriotto et al., 1999; Marciniak and Ron, 2006; Ron and Walter, 2007). When the level of unfolded proteins exceeds the adaptive capacity, intra- and extracellular protein aggregations form, resulting in cellular dysfunction and cell death (Walter and Ron, 2011; Figure 1).

## Neurodegenerative Diseases and ER Stress

Long-term failure of quality control in neuronal cells may cause protein aggregation, leading to neurodegeneration (Ogen-Shtern et al., 2016). Although most neurodegenerative diseases sporadically appear in late-middle age, studies of genetic neurodegeneration suggest that ER stress is related to the onset of conditions.

Protein aggregates called Lewy bodies are found in the brains of patients with Parkinson’s disease (Gibb and Lees, 1988). While most cases are sporadic, causative mutations in α-synuclein and parkin genes have been identified in familial Parkinson’s disease patients (Kalinderi et al., 2016). Mutant α-synuclein aggregates in the cytoplasm and is thought to inhibit proteasomal degradation (Chen et al., 2005). Parkin is an E3-ubiquitin ligase in the ER (Kitada et al., 1998) and promotes the proteasomal degradation of insoluble Pael receptor. Pael receptor is a transmembrane polypeptide. The accumulation of insoluble Pael receptor was observed in Parkinson’s disease patients, which might cause selective neuronal death with ER stress (Imai et al., 2001).

Point mutations of the human Cu/Zn superoxide dismutase 1 (*SOD1*) gene are associated with familial amyotrophic lateral sclerosis (ALS; Rosen et al., 1993), and aggregated SOD1 causes ER stress (Nishitoh et al., 2008). The *C9orf72* gene with hexanucleotide expansions is another major cause of familial ALS (DeJesus-Hernandez et al., 2011; Renton et al., 2011). Motor neurons derived from induced pluripotent stem cells of patients with mutant *SOD1* (Kiskinis et al., 2014) or mutant *C9orf72* (Dafinca et al., 2016) show increased ER stress. Most ALS patients have motor neuron aggregates of TDP-43, an RNA-binding protein. The activation of homeodomain-interacting protein kinase 2 (HIPK2) was reported to promote ER-stress-induced cell death via the IRE1α-ASK1-JNK pathway in TDP-43 proteinopathy in both sporadic and *C9orf72*-related ALS (Lee et al., 2016).

The mutant huntingtin gene that causes Huntington disease has a large number of N-terminal CAG repeats in its base sequence (MacDonald et al., 1993). The gene product of polyglutamine (polyQ) proteins aggregate in the nucleus, but are also found in the cell body and neurites. These aggregates impair proteolysis by the proteasome, causing ER stress and neuronal cell death by the UPR (Nishitoh et al., 2002). The C-terminal fragments that do not contain the polyQ stretch also induce toxicity via dilation of the ER and increased ER stress caused by decreased dynamin 1 function at ER membranes (El-Daher et al., 2015).

Alzheimer disease (AD) is the most common type of dementia and is neuropathologically characterized by intracellular neurofibrillary tangles of tau protein and extracellular senile plaques composed of amyloid β (Aβ). Aβ aggregated outside the cells is considered to cause toxicity with multiple mechanisms such as oxidative stress, mitochondrial dysfunction, synaptic dysfunction and effects on signaling pathways (e.g., insulin, Wnt; Alberdi et al., 2010; Saraiva et al., 2010; Ferreira and Klein, 2011; Arrázola et al., 2015). Early onset AD occurs in patients with autosomal dominantly inherited mutations in the genes encoding amyloid precursor protein (*APP*), presenilin-1 (*PSEN1*), and presenilin-2 (*PSEN2*, Tanzi and Bertram, 2005). APP is cleaved by α-, β-, and γ-secretases; processing by β- and γ-secretases produce Aβ. Certain APP mutations increase Aβ production. PSEN is a part of the γ-secretase complex, and *PSEN1* or -*2* mutations produce more amyloidgenic Aβ42. While Aβ deposition causes ER stress (Pinkaew et al., 2015), mutations in *PSEN1* disturb the UPR and reduce BiP production (Katayama et al., 1999, 2001), suggesting that ER stress is involved in AD onset. Independent of Aβ deposits, intracellular phosphorylated tau and UPR activation have been observed in familial tauopathies (Nijholt et al., 2012).

## Transport in Early Secretory Pathways

Accumulation of misfolded proteins induces the UPR, which affects a wide range of secretory pathways including transport between the ER and other organelles (de Brito and Scorrano, 2010; Vannuvel et al., 2013; Jin et al., 2017).

Intracellular protein trafficking is performed by membrane carriers consisting of coated vesicles and tubular structures. Mature proteins are packaged into coat protein complex (COP) II -coated vesicles and transported from the ER (Schekman and Orci, 1996). The protein is then transferred to the intermediate compartment (IC) and Golgi complex along the microtubules (Bonfanti et al., 1998). Upon arrival at the Golgi, proteins are further sorted into other compartments, while certain proteins are recovered to the ER from the IC or Golgi complex by reverse transport with COPI-coated vesicles (Letourneur et al., 1994). Selective retrograde transport of COPI vesicles is provided by several mechanisms. The carboxyl terminal di-lysine (KKXX) sequence is directly recognized by COPI vesicles (Letourneur et al., 1994) in type I ER membrane proteins such as calnexin (Nilsson et al., 1989). Whereas many ER soluble proteins remain in the ER by interacting with other resident proteins (Raykhel et al., 2007), some soluble proteins are secreted and taken back to the ER. Like BiP, their carboxyl-terminal Lys-Asp-Glu-Leu (KDEL) sequence (Munro and Pelham, 1987) is recognized by the KDEL receptor in the post-ER compartment (Lewis and Pelham, 1992). They are then transported by the COPI vesicle together with the KDEL receptor (Orci et al., 1997). Three human KDEL receptors recognize ER-soluble proteins with KDEL-like motifs (Raykhel et al., 2007).

KDEL receptor 1 also regulates COPI transport (Aoe et al., 1997, 1998). The Ras-like small GTPase ADP-ribosylation factor 1 (ARF1) manages COPI vesicle formation (Rothman and Wieland, 1996). ARFGAP1 induces the hydrolysis of GTP to GDP on ARF1 (Cukierman et al., 1995) and is necessary for proper COPI vesicle formation (Hsu et al., 2009). Notably, ligand binding on the luminal side of KDEL receptor 1 induces its interaction with ARFGAP1 on the cytoplasmic side of the receptor. Therefore, KDEL receptors function as a passive cargo protein for COPI transport and also regulates this pathway via ligand recognition (ER chaperones; Figure 1).

## Retrieval by the KDEL Receptor Plays a Significant Role in the Early Secretory Pathway

BiP is one of the most important ER chaperones and plays central roles in protein folding, degradation, and UPR regulation (Hendershot, 2004). BiP localizes to the ER via interaction with other ER proteins and the ER matrix. As misfolded proteins accumulate in the ER, BiP dissociates from several ER membrane proteins such as inositol requiring kinase-1 (IRE1), PKR-like ER related kinase (PERK), and activated transcription factor 6 (ATF6). BiP dissociation activates these kinases and transcription factors and enhances X-box binding protein-1 (XBP-1) and ATF4 expression, ultimately activating the UPR (Bertolotti et al., 2000). BiP associates with misfolded proteins for refolding. When the complexes are transported from the ER (Hammond and Helenius, 1994), the KDEL receptor 1 recognizes the KDEL sequence of BiP, thus enhancing COPI transport (Yamamoto et al., 2001). We established transgenic mice expressing a mutant KDEL receptor whose reverse transport from the Golgi complex to the ER was restricted. Some of these mice died 10 weeks after birth. Cardiohypertrophy was noted in these animals, and myocardial cell death was observed by terminal deoxyribonucleotidyl transferase (TdT)-mediated biotin-16-dUTP nick-end labeling (TUNEL) assay, which corresponded to dilated cardiomyopathy. Electron microscopy showed protein aggregates in the membrane fraction continuous with sarcoplasmic reticulum. Thus, the retrieval system significantly contributes to protein quality control in the early secretory pathway *in vivo* (Hamada et al., 2004).

## BiP and its Retrieval are Essential in Mammalian Development

Complete deletion of BiP is lethal to yeast cells (the *Kar2* gene in yeast; Rose et al., 1989) and early mouse embryonic cells (Luo et al., 2006). Although nucleotide exchange is essential for substrate binding to BiP (Hendershot et al., 1995, 1996), mutations in the *Sil1* gene that encodes an adenine nucleotide exchange factor for BiP do not cause a lethal phenotype. Mice or humans with homozygous or compound mutations in *Sil1* genes develop various degrees of neurodegeneration (Senderek et al., 2005; Zhao et al., 2005). In fact, another co-chaperone, Grp170, may compensate for Sil1 loss. Interestingly, *Sil1* mutations do not affect the binding of immunoglobulin to BiP (Ichhaporia et al., 2015). While the phenotypes of *Sil1* mutations may reflect the failure of BiP activity to some extent, it depends on its expression in various cell types and developmental stages (Behnke et al., 2015).

**Figure 2:**
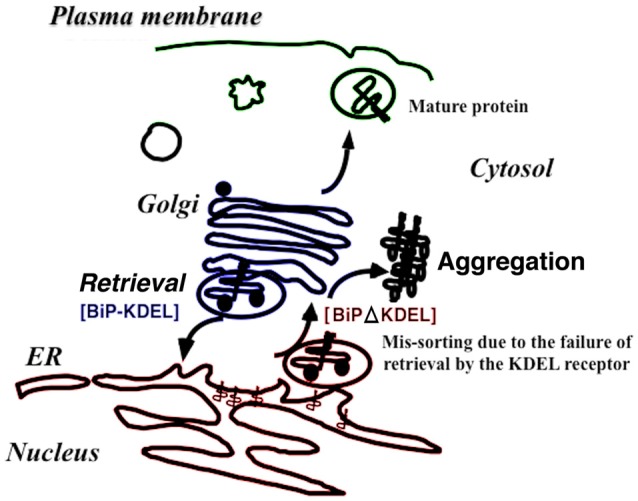
The retrieval system significantly contributes to protein folding quality control in the early secretory pathway *in vivo*. When immunoglobulin heavy-chain binding protein (BiP) is secreted with misfolded proteins from the ER, the Lys-Asp-Glu-Leu (KDEL) sequence of BiP is recognized by the KDEL receptor, resulting in retrieval to the ER via COPI transport. Failure to recover the mutant BiP (ΔKDEL) impairs folding of certain proteins and may cause protein aggregation.

Knock-in mice expressing mutant BiP with the retrieval sequence deleted instead of the wild-type BiP may clarify how BiP retrieval occurs during development and adulthood (Mimura et al., 2007). Homozygous mutant BiP mice are born at full term but die within 1 day because of respiratory insufficiency (Mimura et al., 2007). Neonatal respiratory distress syndrome causes significant mortality. Pulmonary surfactant consists of proteins and phospholipids and is required for proper lung development and function. Compared with wild-type mice, the expression of surfactant protein-A (SP-A) and, more prominently, proSP-C was decreased in the mutant lung. The expression of other components proSP-B and SP-D were unchanged. SP-C remained in the ER, and its expression was significantly reduced in mutant type II pulmonary alveolar cells as evaluated by confocal laser microscopy. ProSP-C is a type II integral membrane protein with structural homology with proteins of the amyloidgenic BRI family that cause neurodegenerative dementia (Kim et al., 2002). ProSP-C tends to misfold and may cause protein aggregation and ER stress (Beers and Mulugeta, 2005). Selective misfolding of proSP-C in mutant BiP type II cells could cause respiratory failure by inducing ER stress in concert with decreased pulmonary surfactant levels (Mimura et al., 2007; Figure 2).

## BiP and its Retrieval Play Important Roles in Neuronal Migration and Neurodegeneration in Aging

Besides respiratory failure, homozygous mutant BiP mice show dysregulated neuronal development (Mimura et al., 2008). Cortical neurons are born in the ventricular zone and move to the marginal zone through older neurons, finally migrating to the final location with an inside-out pattern during cortical development (Caviness, 1982). Conversely, newborn neurons in mutant BiP mice move to the marginal zone and stay there, and later-born neurons cannot migrate to the upper layer. The mutant brain exhibits an outside-in pattern of neocortical layer formation (Mimura et al., 2008). Neuron migration is mediated by the large glycoprotein reelin that is secreted by Cajal-Retzius (CR) cells. In wild-type mice, neurosecretory CR cells are located in the superficial layer of the cortex. However, those in mutant BiP mice are scattered around the upper layer of the neocortical primordium. CR cells in the BiP mutant do not secrete reelin. Although mRNA levels are normal, reelin protein expression is decreased. The formation of cerebellar external granular layer moving tangentially from the rhombic lip was also significantly affected in the mutant BiP mice. Purkinje cells in mutant mice remain in the subcortical region, while they migrate appropriately in wild-type animals (Mimura et al., 2008). Layer formation in the cerebral cortex and cerebellum of the mutant BiP mice were similar to malformations in reeler mutants in which *Reelin* gene is defective (Fatemi, 2001).

Heterozygous mutant BiP mice live to adulthood, with no significant difference in life span compared to wild-type mice. However, potential vulnerability to ER stress may exist in the mutant BiP mice, which develop renal tubular-interstitial lesions as they age (Kimura et al., 2008). While we could not detect significant motor disability in young animals, some of the mutant BiP mice displayed paralysis and tremors after 12 months. They exhibited loss of righting reflex and suffered from paralysis. Motoneurons in the anterior horn of spinal cords of aged mutant BiP mice show evidence of ER stress. Some large motoneurons express C/EBP homologous protein (CHOP), a cell death-related transcriptional factor during ER stress. In accordance with these findings, TUNEL staining identified several apoptotic cells and enhanced gliosis in the mutant spinal cord (Jin et al., 2014). Labeling with an anti-ubiquitin antibody showed cytosolic protein aggregation in large cells at the anterior horn in mutant BiP mice. Experiments using an anti-SOD1 antibody revealed that the perinuclear distribution of SOD1 on large cells was at the anterior horn in wild-type mice, while cytoplasmic aggregations were observed in mutant BiP mice (Jin et al., 2014).

Late-onset AD seems to be sporadic, but the e4 allele of the apolipoprotein E (*APOE*) gene is considered a major risk factor (Wisniewski et al., 1995; Verghese et al., 2011). ApoE is a cholesterol transporter and binds to the APOE receptor type 2 (ApoER2) and very low-density lipoprotein receptor (Vldlr). Reelin also binds these receptors on cortical neurons. Reelin and ApoE4 bind competitively to ApoER2 (Chen et al., 2010). Reelin is secreted by CR cells during neonatal periods to facilitate neuron migration and in the postnatal brain by subtypes of gamma-aminobutyric (GABA)ergic interneurons (Campo et al., 2009). Impaired reelin signaling may be related to the development of psychiatric diseases such as schizophrenia, autism, and bipolar disorder (Tissir and Goffinet, 2003; Fatemi, 2005; Ishii et al., 2016). Reelin signaling has also been suggested to be protective against AD by suppressing Aβ-induced dysfunction of N-methyl-D-aspartate (NMDA) receptors (Durakoglugil et al., 2009). It is interesting that reelin may play important roles in both neuronal development and neurodegeneration and that the folding of reelin is dependent on BiP retrieval.

## The Role of the Retrieval of BiP by the KDEL Receptor

As single cells, embryonic fibroblasts from homozygous BiP mutants survive similar to mutant yeast cells with the *Kar2ΔHDEL* gene (Beh and Rose, 1995). However, experiments using mutant BiP mice revealed that the production of certain complex proteins like reelin and surfactant protein C in secretory cells may require appropriate quality control in the early secretory pathway, including retrograde BiP transport.

The absence of the KDEL sequence from BiP may cause functional impairments in the KDEL receptor, in addition to defective protein folding in the early secretory pathway. Ligand recognition by KDEL receptors transduces the signal from the luminal side to the cytosol, which activates heterotrimeric G-protein signaling (Giannotta et al., 2012), Src kinases (Bard et al., 2003), protein kinase A (Cabrera et al., 2003), and ARFGAP1 for modulation of COPI transport and Golgi apparatus morphology (Aoe et al., 1998; Numata et al., 2013). KDEL receptor 1 also regulates UPR via such as mitogen-activated protein kinase (Yamamoto et al., 2003) and protein phosphatase 1 signaling (Kamimura et al., 2015). Activation of the KDEL receptor 1 promotes autophagy and removal of aggregated proteins including SOD1 (Wang et al., 2011). Loss of the activation may lead to late-onset neurodegenerative diseases or chronic renal diseases. Since BiP is a major ER chaperone that is upregulated during ER stress, KDEL receptor activation by BiP may play a significant role in the early secretory pathway and the pathologies of various diseases.

## ER Stress Research Is Yielding Therapeutic Applications

Sporadic, age-associated neurodegenerative diseases may be due to a failure of quality control machinery in coping with chronic protein aggregation and accumulation. ER chaperone function and expression decrease with age (Brown and Naidoo, 2012). Although UPR activation induces the production of cytoprotective chaperones, it also causes neurotoxicity. Inhibition of IRE1 signaling has been shown to reduce Aβ deposition (Duran-Aniotz et al., 2017). Attempts have been made to increase cell tolerance and reduce UPR overactivation by administering chemical (pharmacological) chaperones that facilitate quality control in the early secretory pathway. Tauroursodeoxycholic acid (TUDCA) is a taurine conjugate of ursodeoxycholic acid (UDCA), a hydrophilic bile acid used to treat cholestatic liver diseases. Both UDCA and TUDCA are potent inhibitors of apoptosis (Vang et al., 2014). 4-Phenylbutyrate (PBA) is a chemical derivative of butyric acid. The effects of PBA are partly due to its ability to regulate gene expression by acting as a histone deacetylase inhibitor and partly because of its stabilizing protein conformation (Kusaczuk et al., 2015). Administration of PBA and TUDCA has been effective in rodent models of diabetes (Ozcan et al., 2006), inflammatory bowel disease (Cao et al., 2013), and familial epilepsy (Yokoi et al., 2015). In a human clinical trial of 34 ALS patients who received TUDCA for 54 weeks, symptom progression was significantly reduced compared to the control group (Elia et al., 2016). Salubrinal is a selective inhibitor of dephosphorylation of phospho-eukaryotic translation initiation factor 2 subunit-α, expanding the general translational repression. Salubrinal protects cells from ER stress, and clinical application is expected (Boyce et al., 2005).

## Conclusion

The KDEL receptors are not merely transport receptors. They regulate the UPR signaling. BiP is a major ER chaperone and contributes to the activation of the KDEL receptor. The first studies of quality control in the early secretory pathway were in yeast, and these results are still guiding clinical research and therapeutic applications in humans. Drugs and gene therapy could one day enhance protein quality control and treat a wide range of diseases.

## Author Contributions

HJ, MK and TA edited the content and structure of the review. TA wrote the review and generated the figures.

## Conflict of Interest Statement

The authors declare that the research was conducted in the absence of any commercial or financial relationships that could be construed as a potential conflict of interest.
